# Racial Variations in Appetite-Related Hormones, Appetite, and Laboratory-Based Energy Intake from the E-MECHANIC Randomized Clinical Trial

**DOI:** 10.3390/nu11092018

**Published:** 2019-08-28

**Authors:** James L. Dorling, Timothy S. Church, Candice A. Myers, Christoph Höchsmann, Ursula A. White, Daniel S. Hsia, Corby K. Martin, John W. Apolzan

**Affiliations:** Pennington Biomedical Research Center, Louisiana State University System, 6400 Perkins Road, Baton Rouge, LA 70808, USA

**Keywords:** energy intake, food intake, satiety, race, exercise, physical activity, health disparities, body composition, satiety quotient, visual analogue scales

## Abstract

African Americans (AAs) have a higher obesity risk than Whites; however, it is unclear if appetite-related hormones and food intake are implicated. We examined differences in appetite-related hormones, appetite, and food intake between AAs (*n* = 53) and Whites (*n* = 111) with overweight or obesity. Participants were randomized into a control group or into supervised, controlled exercise groups at 8 kcal/kg of body weight/week (KKW) or 20 KKW. Participants consumed lunch and dinner at baseline and follow-up, with appetite and hormones measured before and after meals (except leptin). At baseline, AAs had lower peptide YY (PYY; *p* < 0.01) and a blunted elevation in PYY after lunch (*p* = 0.01), as well as lower ghrelin (*p* = 0.02) and higher leptin (*p* < 0.01) compared to Whites. Despite desire to eat being lower and satisfaction being higher in AAs relative to Whites (*p* ≤ 0.03), no racial differences in food intake were observed. Compared to Whites, leptin increased in the 8 KKW group in AAs (*p* = 0.01), yet no other race-by-group interactions were evident. Differences in appetite-related hormones between AAs and Whites exist; however, their influence on racial disparities in appetite, food intake, and obesity within this trial was limited.

## 1. Introduction

Obesity and related comorbidities are a common problem in westernized societies, with minority racial groups being at increased risk. One racial group disproportionally affected by obesity includes African Americans (AAs) [1]. Compared to Whites, AAs have an increased risk of obesity and consequently experience higher rates of diabetes and cardiovascular disease [2,3,4]. 

Numerous studies sought to elucidate the mechanisms underlying these disparities by investigating racial differences in energy homeostasis and associated modifiers. While some studies showed that AAs exhibit lower energy expenditure than Whites [5,6], relatively few investigated racial variations in the physiological control of appetite regulation and food intake. A handful of studies report that, compared to Whites, concentrations of leptin are higher [7,8] and peptide YY (PYY) [7,8] and ghrelin [9,10] are suppressed in AAs. Nevertheless, it is not known if AAs and Whites present different concentrations of glucagon-like peptide-1 (GLP-1), an anorexigenic hormone secreted primarily from intestinal L-cells, akin to PYY [11]. It is additionally unclear if disparities in appetite-related hormones translate into differences in appetite perceptions and food intake. Two studies reported that subjective appetite perceptions in AAs and Whites were similar [9,12], although neither measured energy or macronutrient intake. Thus, it is important to compare race differences in appetite-related hormones in combination with appetite and laboratory-based food intake assessments. Such comparisons would elucidate race-related interconnections between appetite-related hormones and energy intake, providing implications for health disparities related to obesity.

In addition, no study has directly compared the exercise-induced changes in appetite, appetite-related hormones, food intake, anthropometrics, and body composition in AAs and Whites. Some showed that AAs lose less weight than Whites during weight loss regimens that include a change in dietary habits [13], yet the differential effect of exercise alone on weight loss in AAs and Whites is not known. As exercise is often associated with large inter-individual variability in weight loss [14], it is important to examine if race modulates this variability. It is also important to assess whether race alters the change in appetite-related hormones and food intake, since weight loss is a big driver of changes in appetite-related hormones [15].

Our primary aim was to examine differences in appetite-related hormones, subjective appetite perceptions, and food intake between AAs and Whites. We hypothesized that, compared to Whites, AAs would display lower absolute concentrations of leptin, PYY, GLP-1, and ghrelin, as well as blunted postprandial concentrations of PYY, GLP-1, and ghrelin. Our exploratory aim was to examine race differences in appetite-related hormones, appetite, food intake, anthropometry, and body composition in response to a 24-week supervised, controlled aerobic training randomized control trial (RCT). 

## 2. Materials and Methods

### 2.1. Study Design

The present study is a sub-analysis of AA and White participants who completed the Examination of Mechanisms of Exercise-Induced Weight Compensation (E-MECHANIC) RCT (ClinicalTrials.gov: NCT01264406). The Pennington Biomedical Institutional Review Board approved the E-MECHANIC protocol in 2010 (protocol ID: 10008) and data was collected between November 2010 and December 2015 at Pennington Biomedical Research Center (Baton Rouge, LA, USA). All participants provided written informed consent, and a data and safety monitoring board supervised the study. Detailed information about the E-MECHANIC protocol and participant inclusion and exclusion criteria were reported elsewhere [16]. Healthy adults who were overweight or obese (body mass index (BMI): ≥25 kg/m^2^ to ≤45 kg/m^2^), sedentary (not exercising >20 min on ≥3 days/week), not suffering from cardiometabolic disease, and not currently partaking in any weight loss program were recruited. Participants were randomized to one of three groups for 24 weeks: a control group that received health information only, a group that was prescribed an exercise dose recommended for general health (8 kcal/kg of body weight per week (KKW)), or a group that was prescribed an exercise dose that is recommended for weight loss and weight loss maintenance (20 KKW) [16]. Exercise was supervised on a treadmill at a target intensity of 65–85% of peak oxygen uptake (VO2_peak_), with sessions varying in length to adhere to the participants’ energy expenditure goals. Energy expenditure rate was measured to adjust the daily exercise time to account for changes in metabolic or biomechanical efficiency [16]. Participants who identified as AA and White were studied in our analysis. Participants were removed from analysis because of inadequate energy intake (i.e., less than 15 kJ consumed) or because they did not complete the study for various reasons, including loss of contact, family issues, or injury. 

### 2.2. Outcome Variables

#### 2.2.1. Anthropometry and Body Composition

At baseline and follow-up, height and body weight were measured with participants wearing only a weighed clinic gown. Dual-energy X-ray absorptiometry (DXA) (Lunar iDXA and Encore software version 13.60; GE Healthcare, Madison, WI, USA) was used to examine fat mass, fat-free mass, and fat percentage at baseline and follow-up. 

#### 2.2.2. Laboratory Food Intake Tests

Participants visited the laboratory at baseline and follow-up to consume a lunch and dinner meal. Participants were instructed to consume a standardized 190-kcal nutrition bar between 7:00 a.m. and 8:00 a.m. before arriving at the laboratory between 11:00 a.m. and 12:00 p.m. Participants were then provided with an ad libitum lunch test meal that consisted of sandwiches, potato chips, cookies, and water. Moreover, participants were provided an option of artificially sweetened or sugar-sweetened soda or tea [16]. Compared to Whites, a greater proportion of AAs selected sweetened drinks (AAs: 83% vs. Whites: 60%; *p* < 0.01), whereas a lower percentage selected artificially sweetened drinks (AAs: 60% vs. Whites: 79%; *p* = 0.01). Participants returned to the laboratory 5.5 h after the start of the lunch meal to consume a dinner meal. This contained a buffet meal of 18 high- and low-fat foods that were used in a previous study [17]. At lunch and dinner, participants were instructed to consume as much food as they wished. The meals were provided in isolation and participants were blinded to the purpose of food testing. In between lunch and dinner, with the exception of water, participants were instructed not to consume any food or beverages. Participants provided verbal confirmation that they complied with this stipulation. Grams of food and drink consumed were measured using food scales, and energy and macronutrient consumption was quantified using the United States Department of Agriculture’s (USDA) Food and Nutrient Database for Dietary Studies (FNDDS) version 5.0 (ars.usda.gov/ARSUserFiles/80400530/pdf/fndds/fndds5_doc.pdf) [18]. 

Appetite perceptions were collected immediately before and after lunch and dinner through validated visual analogue scales (VASs) [19] on computer-based devices. The questions “how hungry do you feel at this moment”, “how full does your stomach feel at this moment”, “how strong is your desire to eat at this moment”, “how much food do you think you could eat at this moment”, and “how satisfied do you feel at this moment” quantified hunger, fullness, desire to eat, prospective food consumption, and satisfaction, respectively. All VASs were scored 0–100 and contained anchors at either end. Hunger ratings collected after lunch were expressed as the satiety quotient, which measures the strength of hunger suppression caused by ingested food and accounts for variations in nutritional loads [20]. In the current study, the difference in hunger ratings before and after lunch and dinner were expressed relative to the energy consumed. Specifically, satiety quotient = ((hunger before last meal − hunger rating at observation point) × 100)/energy intake consumed up to that moment in the day.

Plasma concentrations of PYY, total ghrelin, and GLP-1 were measured before and after the lunch meal, while serum leptin was measured before lunch only. Whole-blood samples were collected into ethylenediaminetetraacetic acid (EDTA) tubes from participants in a seated position. A protease cocktail (50 µL/mL of whole blood) was added to PYY samples and a dipeptidyl peptidase-IV inhibitor (10 µL/mL of whole blood) was added to GLP-1 collection tubes. Samples were promptly centrifuged at 3000 rpm for 15 min, and plasma fractions were subsequently aliquoted and stored at −80 °C until analysis. Enzyme-linked immunosorbent assays (ELISAs) were used to measure circulating concentrations of GLP-1 (Millipore, Watford, UK), and radioimmunoassays (RIAs) were used to determine PYY, ghrelin, and leptin concentrations (Millipore, Watford, UK). 

### 2.3. Statistical Analysis

Appetite-related hormone concentrations after lunch are presented as delta values to account for day-to-day variability in appetite-related hormones [21], while the area under the curve (AUC) was calculated for satiety quotient using the trapezoidal rule [22]. Non-normally distributed data were log-transformed, yet results did not differ significantly; hence, results from non-transformed data are reported. To examine race differences in appetite-related hormones, appetite, and food intake, general linear models were performed comparing AAs and Whites during the baseline period, with age, sex, and fat mass used as covariates. Energy intake consumed at lunch was an additional covariate for the delta appetite-related hormones. Two-way unpaired general linear models (race × group) examined the change in appetitive measures, anthropometry, and body composition from baseline. Baseline scores were considered a further covariate in these models. Fasting glucose was also included in all analyses, although results were not meaningfully altered; therefore, results without this covariate are reported. Holm–Bonferroni adjusted post hoc *t*-tests were used to locate variance when significant F values occurred. Significance for inferential tests was accepted as *p <* 0.05. Data are displayed as means ± standard error of the mean (SEM), except for descriptive data, which are presented as means ± SD. Analyses were performed using Statistical Package for the Social Sciences (SPSS) version 24 (SPSS Inc., Chicago, IL, USA).

## 3. Results

### 3.1. Participants 

The E-MECHANIC trial enrolled 198 participants at baseline, including 68 AAs and 125 Whites [16]. However, 14 AAs and 13 Whites dropped out, and one AA and one White participant were removed from all analyses for consuming 0 and 13 kJ, respectively, during a lunch meal at the follow-up visit. The final analytic sample was therefore comprised of 53 AA and 111 White participants.

In this sample, AAs were younger than Whites (*p* = 0.02; Table 1). Furthermore, relative VO2_peak_ was lower, while BMI and fat mass were higher in AAs compared to Whites (*p* ≤ 0.01). However, body weight, height, waist circumference, fat-free mass, fat percentage, and fasting blood glucose concentrations were similar between races (*p ≥* 0.06). Unadjusted resting metabolic rate (RMR) did not differ between races (*p* = 0.23), although AAs had lower RMR than Whites after accounting for fat-free mass (*p* = 0.01; Table 1). 

### 3.2. Racial Differences in Appetite-Related Hormones, Appetite, and Food Intake at Baseline

**Figure 1:**
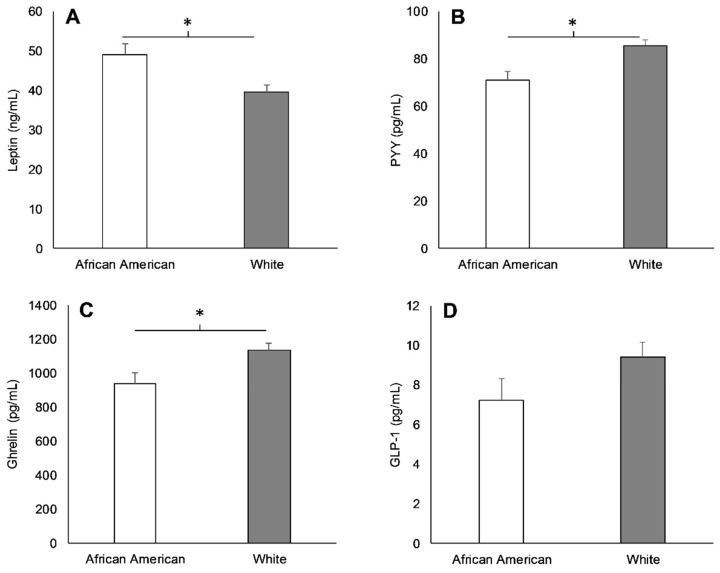
Leptin (**A**), peptide YY (PYY) (**B**), ghrelin (**C**), and glucagon-like peptide-1 (GLP-1) (**D**) in African Americans (AAs) and Whites before lunch during the baseline visit. * Significant difference between races (*p* < 0.05). Values are means ± standard error of the mean (SEM).

**Figure 2:**
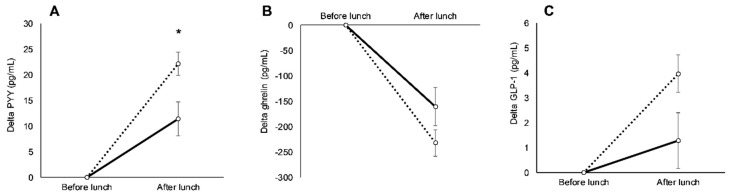
Differences in delta peptide YY (PYY) from before lunch (**A**), delta ghrelin from before lunch (**B**), and delta glucagon-like peptide-1 (GLP-1) from before lunch (**C**) between African Americans (AAs; solid line) and Whites (dashed line) during the baseline visit. * Significant difference between races (mean *p* < 0.05). Values are means ± SEM.

AAs exhibited higher concentrations of leptin (10 ± 3 ng/mL; *p* < 0.01), and lower concentrations of PYY (−15 ± 4 pg/mL; *p* < 0.01) and ghrelin (−192 ± 79 pg/mL; *p* = 0.02) compared to Whites before lunch (Figure 1). There were, however, no differences in GLP-1 levels before lunch in AAs and Whites (−2 ± 1 pg/mL; *p* = 0.11; Figure 1). After lunch, delta PYY concentrations were 11 ± 4 pg/mL lower in AAs relative to Whites (*p* = 0.01), and there was a tendency for lower delta GLP-1 in AAs compared to Whites (−3 ± 1 pg/mL; *p* > 0.05), yet racial differences in delta ghrelin (71 ± 47 pg/mL; *p* = 0.13) were similar (Figure 2). 

**Figure 3:**
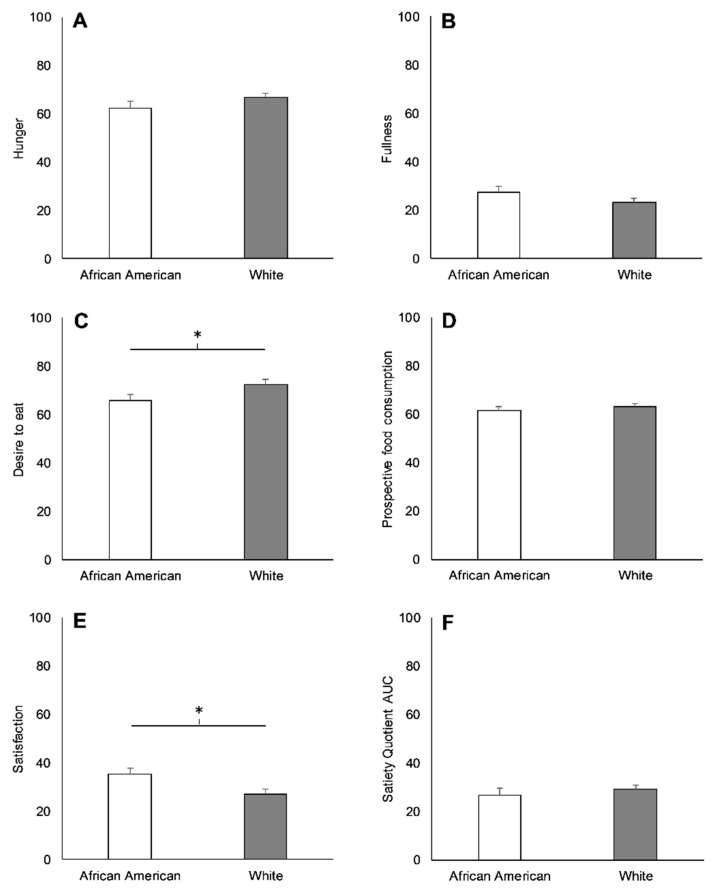
Hunger (**A**), fullness (**B**), desire to eat (**C**), prospective food consumption (**D**), satisfaction (**E**), and satiety quotient area under the curve (AUC) (**F**) in African Americans (AAs) and Whites during the baseline visit. * Significant difference between races (*p* < 0.05). Values are means ± SEM.

Ratings for desire to eat (−7 ± 3; *p* = 0.03) and satisfaction (8 ± 3; *p* = 0.02) were lower and higher, respectively, in AAs than Whites before lunch (Figure 3). Nonetheless, AAs and Whites rated hunger (−4 ± 3; *p* = 0.18), fullness (4 ± 3; *p* = 0.18), and prospective food consumption (−2 ± 2; *p* = 0.46) similarly before lunch, and the satiety quotient AUC after lunch was not different between races (−2 ± 3; *p* = 0.52).

**Figure 4:**
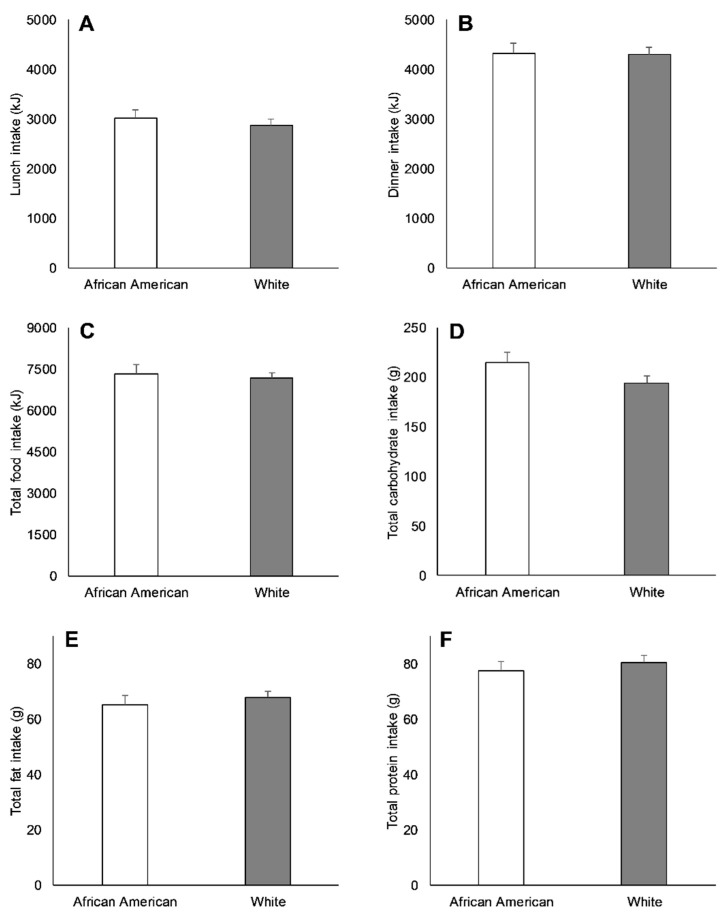
Lunch energy intake (**A**), dinner energy intake (**B**), total energy intake (**C**), total carbohydrate intake (**D**), total protein intake (**E**), and total fat intake (**F**) between African Americans (AAs) and Whites during the baseline visit. No significant differences between races were observed (*p ≥* 0.05). Values are means ± SEM.

At baseline, there were no race-related variations in energy intake at lunch (130 ± 203 kJ; *p* = 0.52), dinner (13 ± 258 kJ; *p* = 0.96), and lunch and dinner combined (168 ± 393 kJ; *p* = 0.67; Figure 4). Likewise, cumulatively, AAs vs. Whites consumed comparable amounts of carbohydrate (21 ± 13 g; *p* = 0.11), fat (−3 ± 4 g; *p* = 0.51), and protein (−3 ± 4 g; *p* = 0.47; Figure 4), with no differences in macronutrient consumption at lunch or dinner separately (Figure S1, Supplementary Materials).

### 3.3. Racial Variations in Appetite-Related Hormones, Appetite, and Food Intake in Response to Exercise

**Figure 5:**
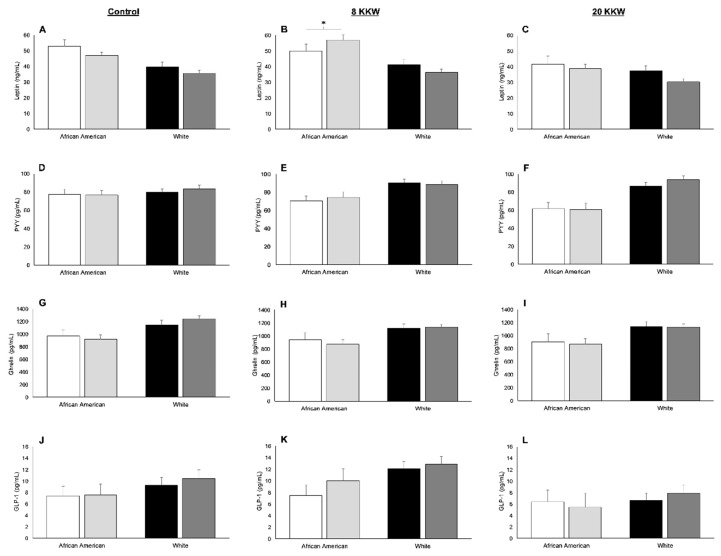
Concentrations of leptin (**A**–**C**), peptide YY (PYY) (**D**–**F**), ghrelin (**G-I**), and glucagon-like-peptide-1 (**J**–**L**) in African Americans (AAs) and Whites at baseline and after the exercise intervention in the control, 8 kcal/kg of body weight/week (KKW), and the 20 KKW groups before lunch. White bars represent African Americans at baseline; light grey bars represent African Americans at the end of the intervention; black bars represent Whites at baseline; dark grey bars represent Whites at the end of the intervention. * Significant two-way interaction between race and group for change score, as examined by a two-way unpaired general linear model, controlling for baseline (*p* < 0.05). Values are means ± SEM.

In response to exercise, there were no main effects of race (*p ≥* 0.11), group (*p ≥* 0.65), or race-by-group interactions (*p ≥* 0.36) for changes in PYY, ghrelin, and GLP-1 concentrations before lunch (Figure 5). However, there was a significant race-by-group interaction for change in leptin (*p* = 0.01). Post hoc analysis demonstrated that the 8 KKW group displayed a 13 ± 4 ng/mL increase in leptin concentrations compared to the control group in AAs (*p* = 0.01) but not Whites (*p* = 0.60; Figure 5).

**Figure 6:**
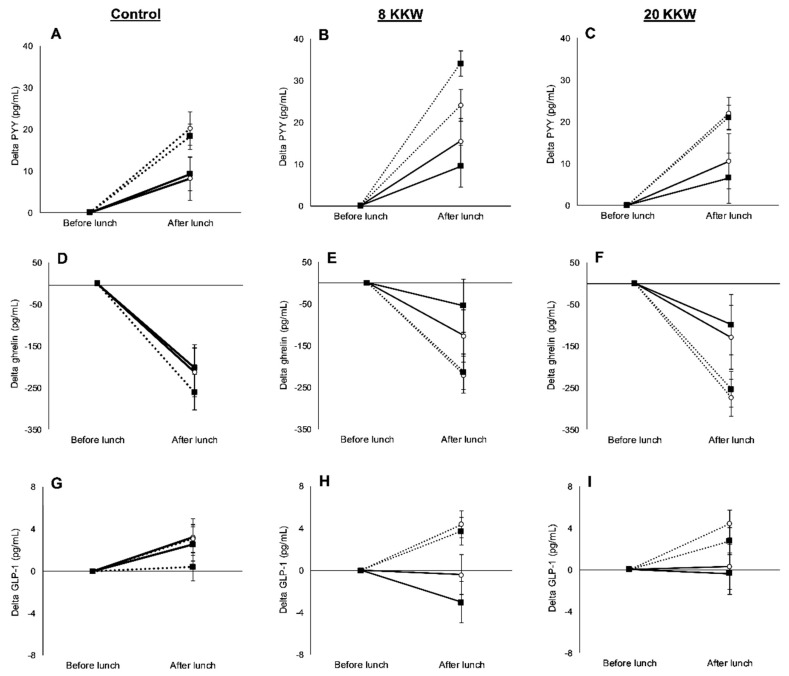
Differences in delta peptide YY (PYY) from before lunch (**A**–**C**), delta ghrelin from before lunch (**D**–**F**), and delta glucagon-like-peptide-1 (GLP-1) from before lunch (**G**–**I**) between African Americans (AAs; solid line) and Whites (dashed line) at baseline (open circle) and after the exercise intervention (dark square) in the control, 8 KKW, and the 20 KKW groups. No significant differences between races and group were observed for change score, as examined by a two-way unpaired general linear model, controlling for baseline (*p ≥* 0.05). Values are means ± SEM.

The changes in delta concentrations of PYY, GLP-1, and ghrelin after lunch were not influenced by race (main effect of race; *p ≥* 0.13), group (main effect of group; *p ≥* 0.43), or by the interaction between race and group (*p ≥* 0.06) in response to exercise (Figure 6).

There was a main effect of race for changes in hunger and satiety quotient AUC, with AAs showing a decrease in hunger (−7 ± 3; *p* = 0.03) and satiety quotient AUC (−11 ± 4; *p* = 0.02) compared to Whites, but changes in other appetite perceptions were similar between races (*p ≥* 0.17; Table 2). Additionally, there was a main effect of group (*p* = 0.03) for prospective food consumption, whereby ratings of prospective food consumption increased in the control group relative to the 20 KKW group (7 ± 3; *p* = 0.03; Table 2). No other group differences were seen (*p ≥* 0.33), and no race-by-group interactions were observed for all appetite perceptions (*p ≥* 0.08; Table 2). 

Changes in lunch energy intake, dinner energy intake, and total energy intake were not affected by race (main effect of race; *p ≥* 0.11), group (main effect of group; *p ≥* 0.11), or a race-by-group interaction (*p ≥* 0.09; Table 2). Apart from protein intake at dinner, where AAs consumed less than Whites after the intervention (main effect of race; *p* = 0.04; Table S1, Supplementary Materials), the changes in lunch, dinner, and total intake of carbohydrate, fat, and protein intake showed no main effects of race (*p ≥* 0.11) and group (*p ≥* 0.08), and no race-by-group interactions (*p ≥* 0.05; Table 2). 

### 3.4. Racial Variations in Anthropometry and Body Composition in Response to Exercise

There were no main race (*p* = 0.96) or race-by-group interaction effects (*p* = 0.30) for weight change (Table 3). A main effect of group was identified for weight change (*p* = 0.04), although there was only a tendency for increased weight loss in the 20 KKW group compared to the control group after post hoc adjustments (20 KKW group: −1.9 ± 0.5 kg vs. control: −0.4 ± 0.4 kg; *p >* 0.05). Change in waist circumference was not influenced by race (main effect of race; *p >* 0.99), group (main effect of group; *p* = 0.23), or a race-by-group interaction (*p* = 0.21; Table 3).

Changes in fat-free mass (*p* = 0.22), fat mass (*p* = 0.47), and fat percentage (*p* = 0.29) were similar in AAs and Whites (main effect of race), and no race-by-group interactions were identified for these measures (*p ≥* 0.36). There was, by contrast, a main effect of group for fat mass (*p* = 0.01), with the 20 KKW group showing a greater loss of fat mass compared to the 8 KKW (−1.2 ± 0.5 kg; *p* = 0.04) and control (−1.6 ± 0.5 kg; *p* = 0.01) groups. Similarly, there was a main effect of group for change in fat percentage (*p* = 0.03). Post hoc comparisons revealed that the 20 KKW group displayed a decrease in fat percentage relative to the control group (−1.0 ± 0.4%; *p* = 0.03).

## 4. Discussion

Our primary objective was to examine racial differences in appetite-related hormones, appetite perceptions, and food intake. Our analysis showed that AAs exhibited higher leptin concentrations, lower concentrations of PYY and ghrelin, and lower desire to eat and higher satisfaction compared to Whites before lunch at baseline. However, no differences were observed for GLP-1 concentrations, and no differences in energy or macronutrient intake were seen during ad libitum test meals. Delta PYY after lunch was blunted in AAs relative to Whites, although changes in ghrelin and GLP-1 were similar between racial groups. As an exploratory aim, we investigated race differences in appetitive measures, anthropometry, and body composition in response to a 24-week aerobic training RCT. Overall, despite AAs in the 8 KKW group showing higher changes in leptin compared to the control group, race and exercise group did not appreciably alter appetite-related hormones in response to the exercise intervention. Race and exercise group also did not interact to affect appetite-related hormones, subjective appetite, or food intake, and both races displayed similar body composition changes during the exercise trial. 

It has been postulated that differences in concentrations of appetite-related hormones may contribute to the greater obesity risk in AAs [12,23], but there is limited evidence that has examined whether racial variations in these hormones translate into meaningful changes in appetitive measures. We found that AAs presented lower concentrations of PYY, further to a blunted elevation in PYY in response to food intake compared to Whites. Attenuated concentrations of PYY are related to reduced satiety [24], yet satiety was not different between AAs and Whites at our baseline visit. Our results also show that AAs had lower desire to eat and higher satisfaction than Whites before lunch, although the mean difference was modest and thus the clinical relevance of these differences could be questioned [25]. In addition to appetite, we saw no differences between AAs and Whites in energy and macronutrient consumption during our test meal day. Some studies indicated that a lower reduction in concentrations of PYY is related to an increase in energy intake and energy-dense foods [21,26], though our results contradict this. Inconsistent associations between appetite-related hormones and appetite and energy intake have been reported [27,28], questioning the influence of appetite-related hormones on food intake. This is supported by studies in humans which showed that appetite-related hormones trigger no changes in appetite or food intake when infused at physiological levels [29,30]. Nevertheless, given the plethora of biological appetite-related agents, it is possible that other appetite-related hormones counteract the variations in PYY between AAs and Whites, meaning appetite and food intake measures remain similar between races [31]. 

Our results suggest that AAs display lower ghrelin concentrations and higher leptin concentrations. Brownley et al. [7] reported that leptin concentrations are higher in AAs, supporting our findings, although studies assessing ghrelin variations in AAs and Whites have been mixed. Some work indicated that lower ghrelin concentrations are evident in AAs compared to Whites [9,10], whereas others showed the contrary [7]. It is possible that contrasting findings are due to discrepancies in controlling body composition, which affects ghrelin concentrations [32]. This could have particular ramifications in AAs as this racial group displays a large degree of variability in body fat percentage for a given BMI [33]. The consequences of lower ghrelin and higher leptin levels in AAs are unclear. However, since resistance to the metabolic actions of both of these hormones is thought to contribute to the development and maintenance of obesity [34], racial disparities in obesity could be influenced by variations in the sensitivity to leptin and ghrelin, with AAs showing lower sensitivity than Whites. Moreover, lower ghrelin and higher leptin levels are associated with poorer regulation of blood glucose regulation, insulin resistance, and lower metabolic flexibility [35,36]. Thus, differences in ghrelin and leptin between AAs and Whites could play a role in the elevated cardiometabolic risk demonstrated by AAs relative to Whites [37].

To our knowledge, our study represents the first study to examine differences in GLP-1 between AAs and Whites. In contrast to other appetite-related hormones, we did not observe any differences in absolute or postprandial concentrations of GLP-1 between AAs and Whites, although there was a tendency for AAs to exhibit lower rises in GLP-1 after lunch. It is possible that the short half-life of GLP-1 makes it difficult to detect differences in concentrations between AAs and Whites from venous blood [11]. One study recently showed that venous concentrations of GLP-1 are smaller and less physiologically relevant from an appetite perspective than those yielded from arterialized blood [38]. As a result, future studies may benefit from performing assessments of arterialized concentrations of GLP-1 to observe differences between AAs and Whites. 

Our assessments of appetite and energy intake collectively infer that variations in appetite-related hormones and satiety are not likely to play a role in the increased obesity risk shown in AAs. It is likely that other physiological disparities, including lower basal metabolic rate and aerobic fitness [39,40], may be more pivotal in explaining the elevated obesity risk in AAs. Indeed, AAs demonstrated suppressed RMR after adjusting for body composition and lower VO2_peak_ values than Whites in our cohort, implying that alterations in energy expenditure and fitness may be more crucial in mediating race-related obesity disparities than differences in appetitive outcomes. Nonetheless, in spite of similar energy and macronutrient intake between races, additional work is needed to examine race-related variations in food and beverage choices, particularly as this and previous work [41] indicated that beverage selection could be different between races.

Intriguingly, in response to exercise, we found that the 8 KKW group showed an increase in leptin concentrations compared to the control group in AAs but not Whites. Leptin concentrations have been shown to decrease during exercise training [42], and this is likely to be driven by exercise-induced reductions in body fat, which is a potent modulator of leptin concentrations [43]. Nevertheless, there were no variations in body fat between the 8 KKW group and control group in response to exercise and no differences between AAs and Whites. The reasons for our race-by-group interaction in leptin concentrations are therefore difficult to interpret, and further studies may be required. In spite of racial differences in leptin, the 8 KKW and 20 KKW exercise interventions engendered similar changes in PYY, ghrelin, and GLP-1 concentrations in AAs and Whites. Furthermore, there were no race-by-group interactions seen for appetite, energy intake, and macronutrient intake, expanding upon previous evidence reporting similar changes in energy intake between AAs and Whites after regimens incorporating dietary changes [13]. We similarly detected no differences between AAs and Whites in body weight changes to exercise. Previous work documented that AAs show attenuated weight loss compared to Whites in interventions that integrate dietary and physical activity modifications; however, these studies induced greater reductions in body fat than were stimulated in our trial [13]. Although this could be expected as exercise per se stimulates small weight loss [44], it is possible that racial differences in appetitive, anthropometric, and body composition outcomes are evident during efficacious weight loss regimens. This may be particularly pertinent for PYY, ghrelin, and GLP-1, since lower adiposity is associated with higher plasma concentrations of these peptides [26,45,46]. 

A strength of our study was the validated measures to determine appetite and energy intake. Our trial was also a randomized controlled trial, with controlled, supervised exercise sessions to ensure the appropriate exercise dose was administered to participants. One limitation of our study is that 73% our sample consisted of women. Consequently, although sex was a covariate in our analysis, our results may primarily be inferred to racial variations in women, indicating that further studies with larger samples of AA and White men are needed. Another limitation is that our study only assessed four appetite-related peptides. Future studies should examine differences in postprandial glucose and other appetite-related hormones such as insulin, oxyntomodulin, amylin, and pancreatic polypeptide between AAs and Whites in order to elucidate how race influences the appetite-related hormone milieu.

## 5. Conclusions

In summary, compared to Whites, AAs display lower concentrations of PYY and a suppressed change in PYY after food intake. Plasma concentrations of leptin and ghrelin were also higher and lower, respectively, in AAs than Whites independent of fat mass. However, variations in laboratory-based energy and macronutrient intake were not seen. We additionally showed that appetitive measures, anthropometry, and body composition were not appreciably affected by the interaction between race and exercise. While additional studies are needed to ascertain the metabolic consequences of racial differences in appetite-related hormones, differences between AAs and Whites in appetite-related hormones appeared not to influence racial disparities in obesity risk in the current trial.

## Figures and Tables

**Table 1:** Demographics, anthropometry, body composition, peak oxygen uptake, and resting metabolic rate in African American (AA) and White Examination of Mechanisms of Exercise-Induced Weight Compensation (E-MECHANIC) participants at baseline.

	African American (AA)	White	*p*-Value
Age (years)	46.0 ± 10.3	50.4 ± 11.7	0.02
Weight (kg)	92.0 ± 13.5	87.1 ± 16.0	0.06
Height (cm)	165.3 ± 6.9	167.7 ± 8.5	0.08
BMI (kg/m^2^)	33.4 ± 4.8	30.6 ± 4.3	<0.01
Waist circumference (cm)	98.7 ± 10.5	99.0 ± 13.3	0.87
Fat-free mass (kg)	48.5 ± 8.3	47.8 ± 10.6	0.69
Fat mass (kg)	40.4 ± 11.4	36.4 ± 8.9	0.01
Fat (%)	45.0 ± 8.3	43.2 ± 6.6	0.13
VO2_peak_ (L/min)	1.9 ± 0.5	2.1 ± 0.6	0.08
VO2_peak_ (mL/kg/min)	21.3 ± 5.0	24.3 ± 5.1	<0.01
RMR (kJ/day)	6163 ± 1042	6422 ± 1381	0.23
RMR ^#^ (kJ/day)	6121 ± 791	6448 ± 791	0.01
Glucose (mg/dL)	91.5 ± 9.7	93.3 ± 6.5	0.15

Abbreviations: BMI, body mass index; VO2_peak_, peak oxygen uptake; RMR, resting metabolic rate. Values are means ± SD. ^#^ Adjusted for fat-free mass.

**Table 2:** Changes in subjective appetite and food intake in response to the 24-week intervention in Africans Americans and Whites.

	African American (AA)			White		
	Control (*n* = 20)	8 KKW (*n* = 20)	20 KKW (*n* = 13)	Control (*n* = 37)	8 KKW (*n* = 38)	20 KKW (*n* = 36)
Hunger before lunch	3 (4) ^a^	−2 (4) ^a^	−1 (5) ^a^	7 (3)	10 (3)	2 (3)
Fullness before lunch	0 (4)	4 (4)	−4 (5)	−3 (3)	−6 (3)	4 (3)
Prospective food consumption before lunch	9 (3)	3 (3)	0 (4) ^b^	6 (2)	7 (2)	0 (2) ^b^
Desire to eat before lunch	9 (4)	1 (4)	2 (5)	4 (3)	7 (3)	3 (3)
Satisfaction before lunch	−12 (4)	−3 (4)	−7 (5)	−3 (3)	−7 (3)	2 (3)
Satiety quotient AUC	−1 (6) ^a^	−9 (6) ^a^	−5 (7) ^a^	10 (4)	8 (4)	1 (4)
Lunch energy intake (kJ)	213 (18)	161 (184)	−174 (221)	−360 (132)	197 (132)	−133 (133)
Dinner energy intake (kJ)	−779 (287)	−783 (299)	−258 (351)	−160 (213)	−299 (210)	−277 (212)
Total energy intake (kJ)	−525 (362)	−861 (370)	−423 (444)	−521 (266)	−120 (264)	−409 (268)
Total carbohydrate intake (g)	−11 (11)	−11 (12)	−7 (14)	−16 (8)	1 (8)	−8 (9)
Total protein intake (g)	−7 (4)	−15 (4)	−9 (5)	−7 (3)	−3 (3)	−6 (3)
Total fat intake (g)	−6 (4)	−11 (4)	−4 (5)	−4 (3)	−3 (3)	−5 (3)

Abbreviations: KKW, kcal/kg of body weight/week; AUC, area under the curve. ^a^ Significant difference between African Americans and Whites (main effect of race; *p* ≤ 0.03); ^b^ significant difference between 20 KKW group and control (main effect of group; *p* = 0.03). Values are means ± standard error of the mean (SEM).

**Table 3:** Changes in anthropometric and body composition measures in response to the 24-week intervention in Africans Americans and Whites.

	African American (AA)			White		
	Control (*n* = 20)	8 KKW (*n* = 20)	20 KKW (*n* = 13)	Control (*n* = 37)	8 KKW (*n* = 38)	20 KKW (*n* = 36)
Weight (kg)	0.2 (0.7)	−0.7 (0.7)	−2.1 (0.8)	−0.9 (0.5)	−0.2 (0.5)	−1.6 (0.5)
Waist circumference (cm)	−0.1 (0.9)	−0.8 (0.9)	−2.3 (1.1)	−1.7 (0.6)	−0.1 (0.6)	−1.4 (0.6)
Fat-free mass (kg)	−0.1 (0.3)	0.0 (0.3)	−0.3 (0.3)	−0.6 (0.2)	−0.2 (0.2)	−0.3 (0.2)
Fat mass (kg)	0.1 (0.6)	−0.9 (0.6)	−2.1 (0.7) ^a^	−0.4 (0.4)	−0.1 (0.4)	−1.4 (0.4) ^a^
Fat percentage (%)	−0.1 (0.4)	−0.5 (0.4)	−1.4 (0.5) ^b^	−0.1 (0.3)	0.0 (0.3)	−0.8 (0.3) ^b^

Abbreviations: KKW, kcal/kg of body weight/week. ^a^ Significant difference between the 20 KKW group and the control and 8 KKW groups (main effect of group; *p* ≤ 0.05); ^b^ significant difference between the 20 KKW group and the control group (main effect of group; *p* ≤ 0.05). Values are means ± SEM.

## Supplementary Materials

The following are available online at https://www.mdpi.com/2072-6643/11/9/2018/s1, Figure S1: Differences in lunch carbohydrate intake, dinner carbohydrate intake, lunch fat intake, dinner fat intake, lunch protein intake, and dinner protein intake between African Americans (AAs) and Whites during the baseline visit. No significant differences between races were observed (*p* ≥ 0.05). Values are means ± SEM, Table S1: Changes in appetite-related hormones and food intake in response to the intervention in Africans Americans and Whites.
