# Supplementary material for: Racial Variations in Appetite-Related Hormones, Appetite, and Laboratory-Based Energy Intake from the E-MECHANIC Randomized Clinical Trial
Source: Nutrients. 2019 Aug 28;11(9):2018. doi: 10.3390/nu11092018 (PMC6770918; doi:10.3390/nu11092018)
Supplement: Supplementary file 1 [file nutrients-11-02018-s001.pdf]

Supplementary files:

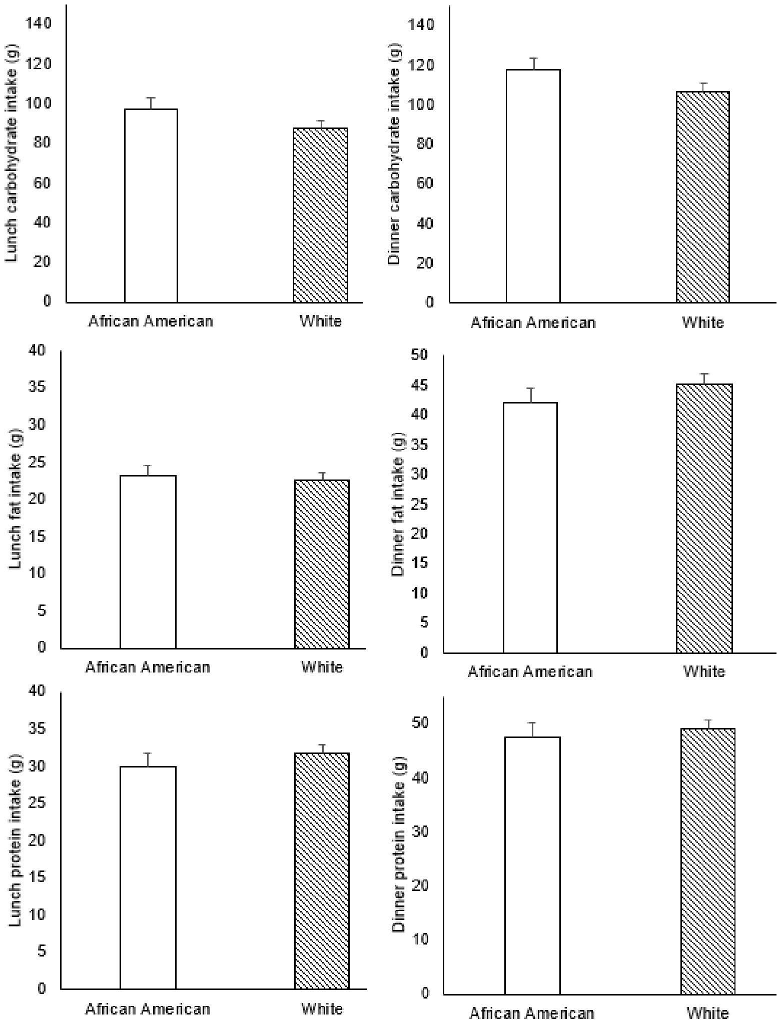

Supplemental Figure 1. Differences in lunch carbohydrate intake, dinner carbohydrate intake, lunch fat intake, dinner fat intake, lunch protein intake, and dinner protein intake between African Americans (AAs) and Whites during the baseline visit. No significant differences between races were observed ($p \geq 0.05$). Values are means ± SEM.

	African American (AA)			White		
	Control (n = 20)	8 KKW (n = 20)	20 KKW (n = 13)	Control (n = 37)	8 KKW (n = 38)	20 KKW (n = 36)
Leptin (ng/mL)	−6 (2)	7 (3)[b]	−3 (3)	−4 (2)	−5 (2)	−7 (2)
PYY (pg/mL)	−1 (5)	4 (6)	−1 (7)	4 (4)	−2 (4)	7 (4)
Delta PYY (pg/mL)	1 (4)	−6 (5)	−4 (6)	−2 (3)	10 (3)	−1 (3)
Ghrelin (pg/mL)	−48 (65)	−64 (68)	−30 (83)	100 (50)	21 (48)	−10 (48)
Delta ghrelin (pg/mL)	10 (56)	72 (64)	30 (72)	−59 (43)	7 (42)	20 (42)
GLP-1 (pg/mL)	0.2 (1.9)	2.5 (2.1)	−0.9 (2.4)	1.2 (1.5)	0.7 (1.4)	1.3 (1.4)
Delta GLP-1 (pg/mL)	−0.7 (1.6)	−2.6 (2.0)	−0.7 (2.0)	−2.6 (1.3)	−0.7 (1.3)	−1.6 (1.3)
Lunch carbohydrate intake (g)	5 (6)	10 (6)	−3 (8)	−12 (5)	4 (5)	−5 (5)
Dinner carbohydrate intake (g)	−16 (9)	−13 (9)	−4 (10)	−5 (6)	−4 (6)	−3 (6)
Lunch fat intake (g)	2 (2)	1 (2)	−3 (2)	−3 (1)	2 (1)	−1 (1)
Dinner fat intake (g)	−9 (3)	−10 (3)	−1 (4)	−1 (2)	−4 (2)	−4 (2)
Lunch protein intake (g)	4 (2)	−2 (2)	−1 (2)	−3 (1)	3 (1)	−1 (1)
Dinner protein intake (g)	−11 (3) [a]	−11 (3) [a]	−8 (4) [a]	−4 (2)	−5 (2)	−5 (2)

Supplemental Table 1. Changes in appetite-related hormones food intake in response to intervention in Africans Americans and Whites.

Abbreviations: KKW, kcal/kg of body weight per week; PYY, peptide YY; GLP-1, glucagon-like peptide. [a] Significant difference between African Americans and Whites (main effect of race; p = 0.04); [b] significant two-way interaction between race and group (p = 0.01). Values are means ± SEM.
